# Comparison of measured electron energy spectra for six matched, radiotherapy accelerators

**DOI:** 10.1002/acm2.12317

**Published:** 2018-03-30

**Authors:** David J. McLaughlin, Kenneth R. Hogstrom, Daniel W. Neck, John P. Gibbons

**Affiliations:** ^1^ Department of Physics and Astronomy Louisiana State University Baton Rouge LA USA; ^2^ Mary Bird Perkins Cancer Center Baton Rouge LA USA

**Keywords:** beam matching, electron energy spectra, magnetic energy spectrometer, percent dose vs depth

## Abstract

This study compares energy spectra of the multiple electron beams of individual radiotherapy machines, as well as the sets of spectra across multiple matched machines. Also, energy spectrum metrics are compared with central‐axis percent depth‐dose (PDD) metrics.

**Methods:**

A lightweight, permanent magnet spectrometer was used to measure energy spectra for seven electron beams (7–20 MeV) on six matched Elekta Infinity accelerators with the MLCi2 treatment head. PDD measurements in the distal falloff region provided *R*_50_ and *R*_80–20_ metrics in Plastic Water^®^, which correlated with energy spectrum metrics, peak mean energy (*PME*) and full‐width at half maximum (*FWHM*).

**Results:**

Visual inspection of energy spectra and their metrics showed whether beams on single machines were properly tuned, i.e., *FWHM* is expected to increase and peak height decrease monotonically with increased *PME*. Also, *PME* spacings are expected to be approximately equal for 7–13 MeV beams (0.5‐cm R_90_ spacing) and for 13–16 MeV beams (1.0‐cm R_90_ spacing). Most machines failed these expectations, presumably due to tolerances for initial beam matching (0.05 cm in *R*_90_; 0.10 cm in *R*_80–20_) and ongoing quality assurance (0.2 cm in *R*_50_). Also, comparison of energy spectra or metrics for a single beam energy (six machines) showed outlying spectra. These variations in energy spectra provided ample data spread for correlating *PME* and *FWHM* with PDD metrics. Least‐squares fits showed that *R*_50_ and *R*_80–20_ varied linearly and supralinearly with *PME*, respectively; however, both suggested a secondary dependence on *FWHM*. Hence, *PME* and *FWHM* could serve as surrogates for *R*_50_ and *R*_80–20_ for beam tuning by the accelerator engineer, possibly being more sensitive (e.g., 0.1 cm in *R*_80–20_ corresponded to 2.0 MeV in *FWHM*).

**Conclusions:**

Results of this study suggest a lightweight, permanent magnet spectrometer could be a useful beam‐tuning instrument for the accelerator engineer to (a) match electron beams prior to beam commissioning, (b) tune electron beams for the duration of their clinical use, and (c) provide estimates of PDD metrics following machine maintenance. However, a real‐time version of the spectrometer is needed to be practical.

## 1 INTRODUCTION

**Figure 1:**
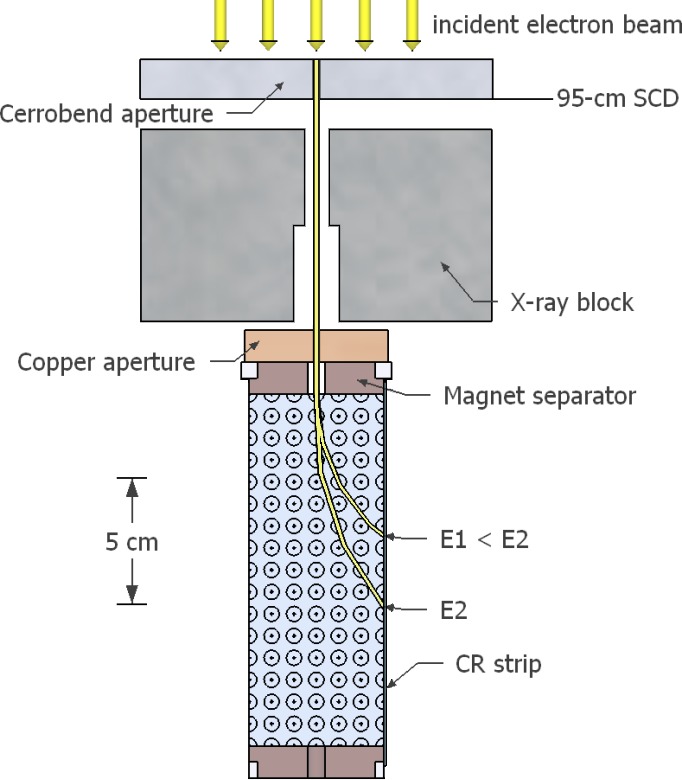
Schematic diagram of top view of central cross‐section of permanent magnet spectrometer. The incident electron beam is collimated into a small circular beam by a Cerrobend^®^ collimator, and a downstream copper aperture defines the circular beam entering the magnet block. The two together create a highly parallel beam and reduce the number of electrons entering the magnet block. The dipole magnetic field (blue), pointing out of the page, bends electrons according to the Lorentz force law, dispersing different energies such that higher energy electrons (E2) travel further downstream than lower energy electrons (E1) before striking the CR strip. The lead x‐ray block shields the CR strip from bremsstrahlung x rays emitted by beamline components. Dimensions are to scale.

Recently, a lightweight, permanent magnet spectrometer and data analysis techniques were developed by McLaughlin et al. for the measurement of energy spectra of therapeutic electron beams.^1^ The 4‐kg spectrometer (16.5 cm long by 5.3 cm wide by 7.8 cm high) contains a dipole, neodymium permanent magnet with a 1.43 cm air separation, producing a 0.54 T field. The magnetic field disperses the energy distributed electrons onto a computed radiography (CR) strip, whose measured spatial distribution transforms to an energy spectrum (cf Fig. 1).

Potential clinical applications of a real‐time version of such a device include, but are not limited to, beam tuning, beam matching, and quality assurance. The aims of the present study were to (a) demonstrate its potential utility for beam matching by comparing electron energy spectra for six matched Elekta accelerators and (b) study the correlation of measured energy spectra metrics with percent depth‐dose curve metrics, showing the potential of the former for estimating percent depth‐dose metrics for quality assurance. Results are reported for a set of seven electron beams on six Elekta Infinity radiotherapy accelerators with the MLCi2 treatment head.

Our institution utilizes matched electron beams, which allow patient treatments to be planned using data for a single machine commissioned on our Pinnacle^3^ (Philips Healthcare, Cambridge, MA) treatment planning system (TPS) and to be treated on any other matched Elekta accelerator. This provides efficiency of medical physicist beam commissioning effort, flexibility in patient machine assignments, and decreased opportunity for treatment error. Our Elekta accelerators, specifically configured for our institution, have seven nominal beam energies (7, 9, 10, 11, 13, 16, and 20 MeV) tuned to have *R*_90_ values of 2.0, 2.5, 3.0, 3.5, 4.0, 5.0, and 6.0 cm (±0.1 cm), which differ slightly from factory‐standard beam tunes. Custom beam energies and our stringent flatness requirements (±3% of central‐axis dose along major axes and ±4% along diagonal axes 2 cm inside the beam edges at depths of 2 cm for *E* > 9 MeV and 1 cm for *E* ≤ 9 MeV)^2^, ^3^ required our matched machines to have dual scattering foils that differ slightly from factory‐standard ones.^4^ The first four of our six Elekta Infinity accelerators utilized the same, modified dual scattering foil systems; whereas, our fifth and sixth accelerators utilized the same modified dual scattering foil systems for 7–13 MeV beams, but a slightly thicker secondary scattering foil for the 16 and 20 MeV beams for improved flatness. Our machines have factory‐standard Elekta electron applicators and matched x‐ray jaw settings, set to ensure acceptable beam flatness and leakage within IEC standards.^5^

Percent depth‐dose curve metrics used for beam tuning during the beam matching process at our institution were *R*_90_ and $R_{80-20}\left(R_{20}-R_{80}\right)$. For the reference machine, beams were tuned such that their *R*_90_ values agreed within 0.05 cm of desired clinical values; subsequently, matched beams were tuned such that their *R*_90_ values agreed within 0.05 cm of reference machine values. Simultaneously, for the reference machine, beams were tuned for minimal *R*_80–20_, and matched beams were tuned such that their *R*_80–20_ values agreed within 0.10 cm. In our clinical experience, matched depth‐dose falloff metrics, scattering foils, and x‐ray jaw settings result in percent dose vs depth, off‐axis ratios, and output factors being matched to within 2% or 0.1 cm.

Elekta electron beams are of particular interest for this study because of their sensitivity to beam tuning, especially recirculated radiofrequency (RF) power. Unusually shaped, multipeak electron energy spectra were reported by Deasy et al.^6^ for a Philips accelerator (Elekta predecessor). Kok and Welleweerd^7^ showed how the unusual shape of these spectra can be attributed to the phase of recirculated RF power. Our measurements were a random ‘snapshot’ of the energy spectra. They indicated that our institution's energy spectra were mostly, but not always, single peaked, apparently a result of good, but not always optimal, beam‐tuning procedures and of beam tune drifting. Our experiences are that Elekta electron beams require frequent beam tuning, particularly the higher energy electron beams.

Hence, we believe the availability of a real‐time electron energy spectrometer would be of value to the accelerator engineer for beam tuning and matching. Also, it might provide reasonable estimates of central‐axis percent depth‐dose (PDD) curve metrics, determining them from energy spectra metrics.

Therefore, this work first compares measured electron energy spectra for the seven beam energies on the six matched electron machines at our institution. Second, it reports on the correlation between measured electron beam energy spectra and central‐axis percent depth‐dose metrics.

## 2 MATERIALS AND METHODS

### 2.A Measurement of energy spectra for seven electron beams on six matched machines

Energy spectra were measured for all seven electron beams on each of our institution's six matched Elekta Infinity radiotherapy accelerators with the MLCi2 treatment head and standard electron applicators, whose downstream surfaces are 5 cm upstream of isocenter, i.e., 95‐cm source‐to‐collimator distance (SCD). For each machine, energy spectra were measured for all energies on a single day using the permanent magnet spectrometer previously fabricated by Rice University (Houston, TX) and described by McLaughlin et al.^1^ The energy spectra were measured for that portion of the electron beams on central‐axis at 95‐cm SCD, which passes through a 0.278‐cm diameter aperture in a 1.59‐cm thick Cerrobend collimating insert placed in the 14 × 14 cm^2^ applicator (cf Fig. 1). The electrons passed downstream through a second 0.318‐cm diameter pinhole copper collimator after which the magnetic field bent the electrons onto a CR strip, which recorded intensity vs position. Subsequent readout of the CR strips produced intensity vs position curves that were converted into energy spectra using methods previously described by McLaughlin et al.^1^ All energy spectra plotted in this study were normalized to have an area of unity.

As energy spectra for most beam energies were closely matched, metrics were used for a more quantitative comparison. We used peak mean energy (*PME*), full‐width at half maximum (*FWHM*), and their ratio *FWHM*/*PME*. *PME*, as defined by McLaughlin et al., is essentially the mean energy over a 30% energy window around the peak.^1^

The precision of energy spectra measurements was estimated by repeating measurements seven consecutive times for the 7, 11, and 16‐MeV beams. The resulting spectra, plotted in McLaughlin,^8^ closely replicated each other. This is reflected in the comparison of *PME* and *FWHM* metrics for each of the seven measurements, which showed a relative uncertainty (one standard deviation) of approximately 0.4% for *PME* and 1.4% for *FWHM*.

### 2.B Measurement of percent depth‐dose metrics

Matched electron beams require energy spectra sufficiently matched to produce matched central‐axis percent depth‐dose curves. In the present study, we evaluated the agreement between *PME* and *FWHM* of the energy spectra necessary for *R*_50_ values to agree within 0.05 cm and $R_{80-20}\left(R_{20}-R_{80}\right)$ values to agree within 0.10 cm for matched machines. To minimize the effect of any day‐to‐day drifting of beam tunes, *R*_50_ and *R*_80–20_ values were measured on the same day that the energy spectra were measured.

*R*_50_ and *R*_80–20_ measurements were made in Plastic Water^®^ phantom slabs (CIRS Inc., Norfolk, VA) to minimize depth inaccuracies due to surface determination in a water phantom and to eliminate the time of setting up a beam scanner and water tank. Our measurement technique paralleled that of our clinic's monthly QA protocol for verifying the constancy of percent depth ionization at a depth near *R*_50_ to within 0.2 cm.^9^, ^10^ These measurements (near *R*_100_ and *R*_50_) along with additional measurements near depths of 80% and 20% ionization, were used to determine *R*_50_ and *R*_80–20_.

Relative ionization was measured with a 0.6 cm^3^ Farmer chamber (TN 30013, PTW, Freiburg, Germany) having a cavity radius, *r*_*cav*_, of 0.3 cm. Measured ionization values at the four depths were normalized to the maximum ionization. Percent ionization vs effective depth (depth minus 0.5*r*_*cav*_) points were converted into percent dose (%*D*) vs depth (*d*) points following AAPM TG‐25 protocol^3^ and its TG‐70 supplement^11^ with TG‐51 values for relative stopping powers,^12^ as implemented in the IBA data acquisition system (IBA, Louvain‐la‐Neuve, Belgium). Lastly, *R*_80_, *R*_50_, and *R*_20_ were determined from a nonlinear, least‐squares fit to the four (%*D*,* d*) points in the falloff region using

(1) $$\%D\left(d\right)=\left(100\%-D_{x}\right)\frac{\text{erfc}(a_{1}d+a_{2})}{2}+D_{x},$$

where *d* is the effective depth, *D*_*x*_ is the energy‐dependent bremsstrahlung dose percent at *R*_*p*_ **+ 2 cm, erfc is the complimentary error function, and *a*_1_ and *a*_2_ are parameters determined by the fit using the nonlinear, Levenberg‐Marquardt algorithm option in MATLAB (MathWorks, Natick, MA). Corrections to %*D* vs depth due to small differences in stopping and scattering powers between water and Plastic Water^®^ were ignored in the present study. Resulting differences in *R*_50_ and *R*_80–20_ would be small, but more importantly would vary smoothly with energy, having insignificant impact on our conclusions.

## 3 RESULTS AND DISCUSSION

### 3.A Comparison of energy spectra from matched beams

**Figure 2:**
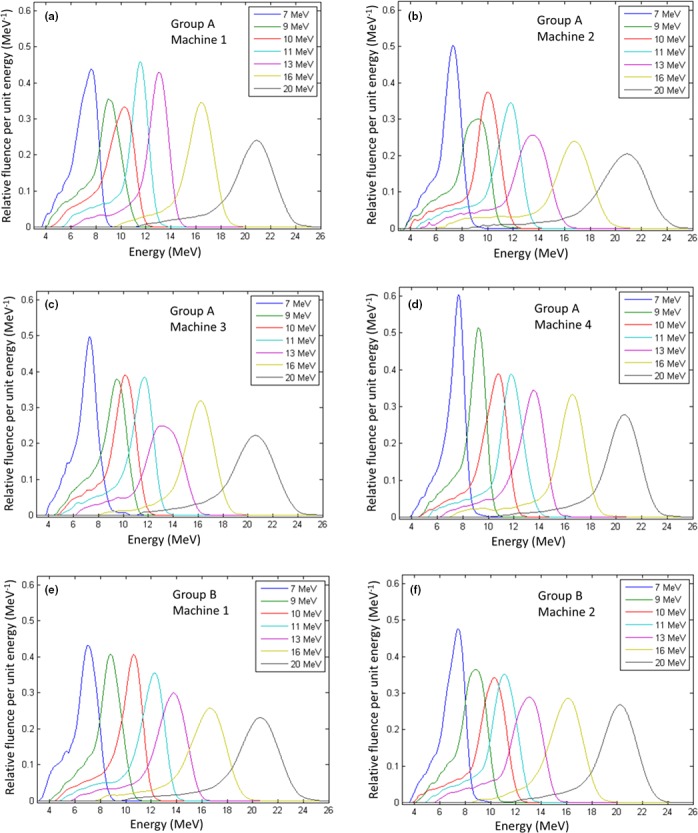
Measured energy spectra for seven electron beams on six matched Elekta Infinity accelerators. (a–d): Four accelerators in group A with beams for machines A‐2 to A‐4 matched to beams of machine A‐1. (e–f) Two accelerators in group B with beams for machine B‐2 matched to beams of machine B‐1. Data are plotted in time order of machine installations.

**Figure 3:**
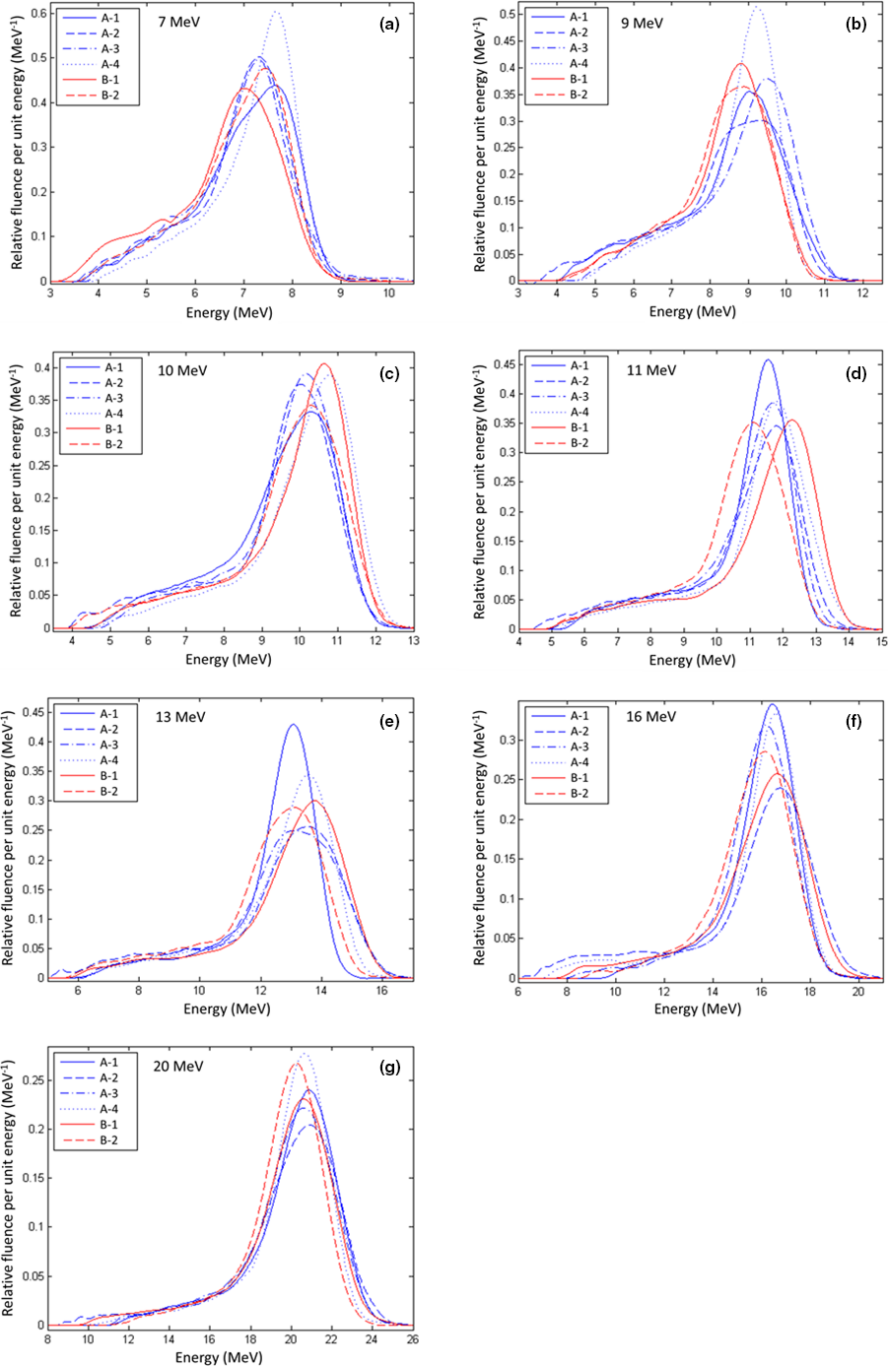
Comparison of measured energy spectra from the six matched Elekta Infinity accelerators for each beam energy from Fig. 2. Plotted are (a) 7 MeV, (b) 9 MeV, (c) 10 MeV, (d) 11 MeV, (e) 13 MeV, (f) 16 MeV, and (g) 20 MeV energy spectra for each of the six matched Elekta Infinity accelerators. The energy spectra from the reference accelerators (A‐1 and B‐1) are plotted as solid lines along with the other energy spectra from group A (blue) and the group B (red).

Figure 2 plots the seven energy spectra (7–20 MeV) for each of the six matched machines (Elekta Infinity radiotherapy accelerators). Machines A‐2 to A‐4 were matched to machine A‐1 (group A), and machine B‐2 was matched to machine B‐1 (group B) at the time of beam setup and commissioning. To visualize the matching of beams of the same energy from different machines, Fig. 3 plots corresponding energy spectra from each of the six machines (1–6) for each of the seven beam energies. The energy spectra for each machine should be approximately evenly spaced for the 7, 9, 10, 11, and 13 MeV beams, tuned to have *R*_90_ values (±0.05 cm) spaced every 0.5 cm (2.0, 2.5, 3.0, 3.5, and 4.0 cm), and for the 13, 16, and 20 MeV beams, tuned to have *R*_90_ values spaced every 1.0 cm (4.0, 5.0, and 6.0 cm). Also, a general trend of increasing width with correspondingly decreasing amplitude of energy spectra with increasing energy is expected according to ICRU Report 35 (eq. 3.15),^13^ as higher energy electron beams typically (a) require thicker scattering foils to broaden and flatten the beams, which cause increased energy loss and hence increased energy straggling and (b) have greater energy spread incident on the scattering foils due to a fixed‐size energy slit in the achromatic bending magnet.

The energy spectra for the seven electron beams (7–20 MeV) for each of the six matched radiotherapy machines had exceptions to these trends. Visually, machine B‐1, the reference machine for group B, shows the energy spectra most exemplary of properly tuned beams, i.e., being uniformly spaced and having increasing spectrum widths and decreasing amplitudes with increasing energy. Contrarily, for machine A‐1 the amplitudes for the 7, 9, and 10 MeV energy spectra are inconsistent with the amplitudes for the 11, 13, 16, and 20 MeV energy spectra. For machine A‐2, the width of the 9 MeV beam is greater than that at 10 MeV, not monotonically decreasing with decreasing energy, as otherwise expected. For machine A‐3, the position of the 9 MeV beam is not midway between the 7 and 10 MeV beams, as otherwise expected, and the *FWHM* of the 13 MeV spectrum is abnormally large. Machine A‐4 energy spectra look almost as expected; however, the 10 MeV beam could have a slightly narrower energy spectra with a slightly greater amplitude. For machine B‐2, the spectra at 9 and 10 MeV also could have had a slightly narrower width with slightly greater amplitude.

Ideally, each spectrum should appear as a single, asymmetric peak,^13^ which is approximately Gaussian‐shaped on the high energy side of the peak and Lorentzian‐shaped on the low energy side. However, it is well known that the energy spectrum can be multipeaked if the recirculated RF power is not in proper phase.^7^ Inspection of our data shows that only a few spectra hinted at being multipeaked, e.g., 7 and 10 MeV for machine A‐1, 9 MeV for machine A‐2, 13 MeV for machines A‐2 and A‐3, and 20 MeV for A‐2.

Because most beams were well matched, a more quantitative comparison that utilizes previously defined peak mean energy (*PME*), full‐width at half maximum (*FWHM*), and their ratio (*FWHM*/*PME*) is given in Table 1. Ideally, the matching energy spectra from different machines would be identical; such is not the case, because (a) each accelerator will tune slightly differently and (b) quality assurance standards^9^, ^10^ allow *R*_50_ to vary ±0.2 cm in water, corresponding to approximately ±0.5 MeV in *PME*. Hence, peak mean energies should fall within a band of 1.0 MeV. Variations in the *PME* from the six matched machines ranged from 0.40 to 0.94 MeV, the largest value due to machine B‐2 at 11 MeV appearing improperly tuned. Variations in *FWHM* for the six matched machines ranged from 0.59 to 1.17 MeV. These spreads in the data were sufficient for studying their correlation to percent depth‐dose metrics.

**Table 1:** Comparison of energy spectra metrics from the six matched Elekta Infinity accelerators for each of the seven beam energies. Metrics are peak mean energy (*PME*), full‐width at half maximum (*FWHM*), and relative width (*FWHM*/*PME*). Far right column lists the difference (Δ) in maximum and minimum values for *PME* and *FWHM* for the six matched machines

Beam	Metric	Group A				Group B		Δ
		A‐1	A‐2	A‐3	A‐4	B‐1	B‐2	
7 MeV	*PME* (MeV)	7.28	7.17	7.10	7.38	6.98	7.15	0.40
	*FWHM* (MeV)	2.23	1.64	1.71	1.64	1.85	1.99	0.59
	*FWHM/PME*	0.31	0.23	0.24	0.22	0.26	0.28	
9 MeV	*PME* (MeV)	9.01	8.98	9.22	8.97	8.70	8.68	0.54
	*FWHM* (MeV)	2.15	2.73	2.34	1.75	2.01	2.32	0.98
	*FWHM/PME*	0.24	0.30	0.25	0.19	0.23	0.27	
10 MeV	*PME* (MeV)	9.93	9.95	9.98	10.41	10.30	10.12	0.48
	*FWHM* (MeV)	2.66	2.05	2.15	2.35	2.21	2.39	0.61
	*FWHM/PME*	0.27	0.21	0.22	0.23	0.21	0.24	
11 MeV	*PME* (MeV)	11.28	11.45	11.33	11.70	11.92	10.98	0.94
	*FWHM* (MeV)	1.78	2.40	2.29	2.07	2.40	2.33	0.62
	*FWHM/PME*	0.16	0.21	0.20	0.18	0.20	0.21	
13 MeV	*PME* (MeV)	12.79	13.35	13.22	13.21	13.47	12.79	0.68
	*FWHM* (MeV)	2.00	3.04	3.38	2.59	2.85	2.86	1.04
	*FWHM/PME*	0.16	0.23	0.26	0.20	0.21	0.22	
16 MeV	*PME* (MeV)	16.13	16.54	15.94	16.29	16.31	15.80	0.74
	*FWHM* (MeV)	2.61	3.26	2.80	2.49	3.42	3.20	0.93
	*FWHM/PME*	0.16	0.20	0.18	0.15	0.21	0.20	
20 MeV	*PME* (MeV)	20.50	20.45	20.30	20.28	20.27	19.92	0.58
	*FWHM* (MeV)	3.74	4.34	4.01	3.17	3.80	3.37	1.17
	*FWHM/PME*	0.18	0.21	0.20	0.16	0.19	0.17	

### 3.B Correlation of beam energy spectra with percent depth‐dose curve metrics

Results comparing percent depth‐dose metrics, *R*_50_ and *R*_80–20_, of matched beams for all seven energy and six machine combinations are listed in Table 2. Most *R*_50_ values were well outside our 0.05 cm tolerances for matched beams because the beams, which previously had been matched during commissioning, were now in clinical use, where the QA tolerance for *R*_50_ is 0.2 cm of their reference values.^10^ Variations in *R*_50_ for the six matched machines ranged from 0.14 to 0.37 cm, the largest value again due to machine B‐2 at 11 MeV apparently being improperly tuned. Variations in *R*_80–20_ for the six matched machines ranged from 0.077 to 0.112 cm. These spreads in percent depth‐dose data were sufficient for correlating to energy spectrum metrics. Hence, the results in Tables 1 and 2 provided a robust data set for correlating values of energy spectra metrics, *PME* and *FWHM*, with values of percent depth‐dose metrics, *R*_50_ and *R*_80–20_.

**Table 2:** Comparison of percent depth‐dose metrics from the six matched Elekta Infinity accelerators for each of the seven beam energies. Metrics are *R*_50_ and *R*_80–20_. Far right column lists the difference (Δ) in maximum and minimum values for *R*_50_ and *R*_80–20_ for the six matched machines

Beam	Metric	Group A				Group B		Δ
		A‐1	A‐2	A‐3	A‐4	B‐1	B‐2	
7 MeV	*R*_50_ (cm)	2.908	2.802	2.868	2.921	2.810	2.777	0.14
	*R*_80–20_ (cm)	1.053	0.994	1.073	1.039	1.021	1.029	0.079
9 MeV	*R*_50_ (cm)	3.593	3.516	3.716	3.573	3.475	3.391	0.32
	*R*_80–20_ (cm)	1.268	1.252	1.288	1.206	1.199	1.194	0.094
10 MeV	*R*_50_ (cm)	4.073	4.030	4.062	4.201	4.157	4.048	0.17
	*R*_80–20_ (cm)	1.328	1.243	1.270	1.308	1.307	1.313	0.085
11 MeV	*R*_50_ (cm)	4.643	4.607	4.684	4.780	4.845	4.475	0.37
	*R*_80–20_ (cm)	1.448	1.462	1.502	1.525	1.520	1.433	0.092
13 MeV	*R*_50_ (cm)	5.278	5.365	5.440	5.414	5.479	5.228	0.25
	*R*_80–20_ (cm)	1.654	1.719	1.721	1.651	1.704	1.644	0.077
16 MeV	*R*_50_ (cm)	6.675	6.678	6.678	6.715	6.649	6.542	0.17
	*R*_80–20_ (cm)	2.056	2.012	1.997	1.970	2.077	2.042	0.107
20 MeV	*R*_50_ (cm)	8.290	8.278	8.333	8.224	8.245	8.119	0.21
	*R*_80–20_ (cm)	2.764	2.798	2.833	2.701	2.780	2.686	0.112

AAPM Report of Task Group 25 recommends the correlation ${\overset{¯}{E}}_{o}=2.4\cdot R_{50}$ for the initial mean energy ${\overset{¯}{E}}_{o}$ in MeV and *R*_50_ in cm;^3^ therefore, in the present study, we linearly correlated *PME* with *R*_50_, i.e.,

(2) $$R_{50}=b_{1}\mathrm{PME}+b_{2}.$$

**Figure 4:**
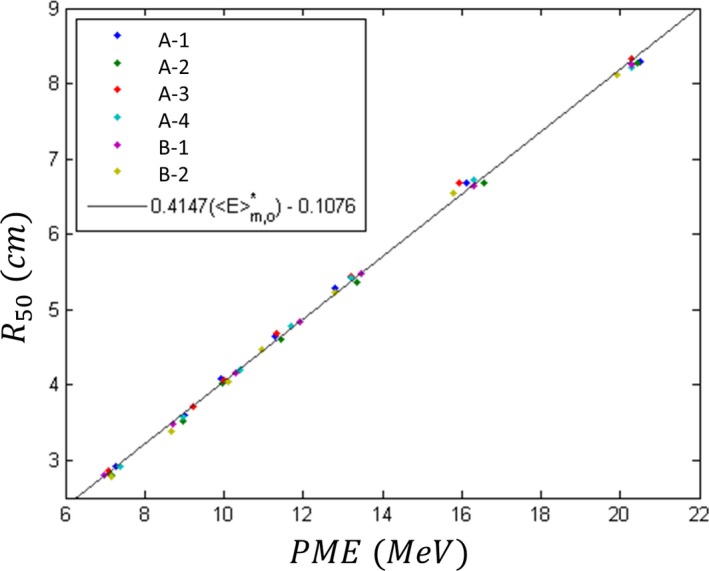
Plot of *R*_**50**_ vs peak mean energy (*PME*). Measured data (colored points) are plotted for the seven beam energies on all six matched Elekta Infinity accelerators. The solid line is the result of fitting *R*_50_ = *b*_1_ *PME* + *b*_2_ to the data (*b*_1_ = 0.4147 cm · MeV^−1^ and *b*_2_ = 0.1076 cm; *R* ^2^ = 0.9987).

Figure 4 plots results of the measured data and fit of eq. (2), which resulted in $b_{1}=0.4147\;\text{cm}\cdot {\text{MeV}}^{-1}={\left(2.41\;\text{MeV}\cdot {\text{cm}}^{-1}\right)}^{-1}$, consistent with AAPM TG25 recommendation, and *b*_2_ = 0.1076 cm. In matching electron beams, our criterion for *R*_50_ is 0.05 cm; therefore, if matching the beam using the measured energy spectrum, the *PME* value should agree to within 0.12 MeV (0.05 cm × 2.4 MeV · cm^−1^). However, the 16 MeV beams have data points that vary from the fit by ±0.3 MeV while agreeing to within 0.05 cm in terms of their *R*_50_ values. Hence, exceeding the 0.12 MeV match criterion for *PME* values does not necessarily mean that *R*_50_ values do not match. This suggests that *PME* alone is not a sufficient surrogate and that *FWHM* also plays a role, contrary to Deasy et al.^14^ For example, the machines A‐2 and A‐3 have *PME* values of 16.54 and 15.94 MeV, respectively, with identical *R*_50_ values of 6.68 cm; however, their *FWHM* values are 2.80 and 3.26 MeV, respectively. This is consistent with results reported by Johnsen et al.^15^

**Figure 5:**
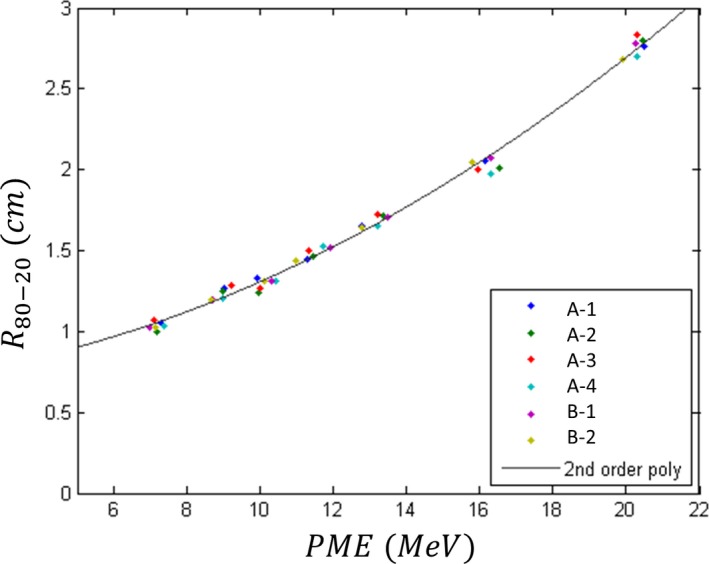
Plot of *R*_**80–20**_ vs peak mean energy (*PME*). Measured data (colored points) are plotted for the seven beam energies on all six matched Elekta Infinity accelerators. The solid line is the result of fitting *R*_**80–20**_ = *a*_**1**_ *PME* ^2^ + *a*_**2**_ *PME* + *a*_**3**_ to the data (*a*_**1**_ = 0.00384 cm · MeV^−2^, *a*_**2**_ = 0.0233 cm · MeV^−1^, and *a*_**3**_ = 0.690 cm; *R* ^2^ = 0.9939).

*R*_80–20_ values for all seven beam energies on all six Elekta Infinity accelerators are plotted vs their respective *PME* values in Fig. 5. A second‐order polynomial, least‐squares fit to these data demonstrates that *R*_80–20_ is primarily governed by the incident *PME*, increasing supralinearly with increasing *PME* values. This is attributed to increased range straggling with increasing *PME*. However, variations among the six data points for each of the seven nominal energies indicate an additional, second‐order dependence on another factor, which almost certainly is the difference in the widths of the energy spectra (*FWHM*). This is also evident for the Atomic Energy of Canada Limited (AECL) Therac 20 and 25 scanned electron beams, which having a much narrower energy spectra (smaller *FWHM*), have substantially smaller values for *R*_80–20_ (cf ICRU 35,^13^ Pfalzner and Clarke,^16^ O'Brien et al.^17^).

**Figure 6:**
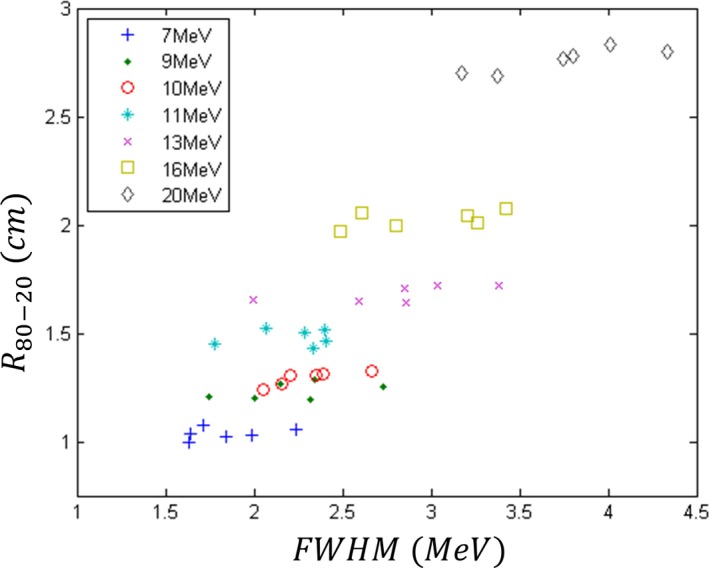
Plot of *R*_**80–20**_ vs *FWHM*. Measured data (colored points) are plotted for the seven beam energies on all six matched Elekta Infinity accelerators. For each beam energy, the data illustrate a slight increase in *R*_**80–20**_ with *FWHM*.

*R*_80–20_ values for all seven beam energies on all six Elekta Infinity accelerators are plotted vs *FWHM* in Fig. 6. For each nominal energy, the *R*_80–20_ value had a second‐order relationship with spectral width, increasing slightly with increases in *FWHM*. For example, for the 10 MeV beam there is a slope of about 0.05 cm · MeV^−1^. Hence, these data confirm that the *FWHM* of the energy spectra plays a minor, but important role in beam matching. Variations from a straight line fit at each energy were due in part to variations in *PME* values for all six accelerators at the same nominal energy. To better understand these data, they were fit to a theory that relates the slope of the dose falloff region with *PME* and *FWHM*. The theory used to relate *R*_80–20_ to *PME* and *FWHM* was a modified version of eq. (6.35) in ICRU 35),^13^, ^18^ i.e.,

(3) $$R_{80-20}\left(PME,FWHM\right)=R_{80-20}\left(\mathrm{PME},0\right)\left[1+c_{1}\cdot \frac{\mathrm{FWHM}}{\mathrm{PME}}\right],$$

where *R*_80–20_ at *PME* for *FWHM* = 0 is modeled by

(4) $$R_{80-20}\left(\mathrm{PME},0\right)=c_{2}\cdot {\mathrm{PME}}^{2}+c_{3}\cdot \mathrm{PME}+c_{4}.$$

**Figure 7:**
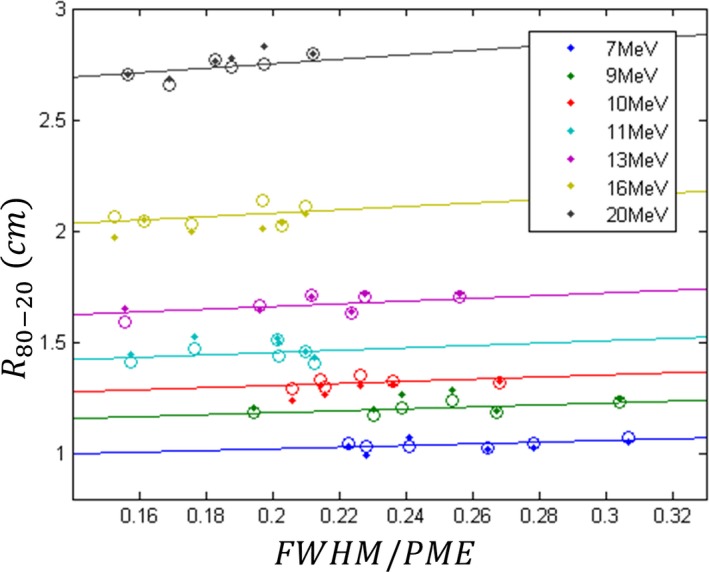
Plot of *R*_80–20_ vs *FWHM*/*PME*, the ratio of *FWHM* to peak mean energy. Measured data (colored dots) are plotted for the seven beam energies on all six matched Elekta Infinity accelerators. Also plotted as open circles are results of fitting eq. (3) to the measured data. The solid lines result from the same fit with *PME* being set equal to the average *PME* for all six data points within a single nominal beam energy and the *FWHM* being allowed to vary, demonstrating the linear dependence of *R*_80–20_ on *FWHM*.

The values for *c*_1_, *c*_2_, *c*_3_, and *c*_4_ were 0.394 ± 0.174, 0.003205 ± 0.000385 cm · MeV^−2^, 0.03414 ± 0.0094 cm · MeV^−1^, and 0.540 ± 0.071 cm, respectively. These values were determined by least‐squares fitting eq. (3) to all the *FWHM* and *PME* values in Table 1 and the corresponding *R*_80–20_ values in Table 2 using the nonlinear, Marquardt algorithm option in the software package ProStat (Pearl River, NY). Plotted in Fig. 7 are all measured data as well as calculated values from the fit of eq. (3) to the data. Figure 7 shows excellent agreement (sum of least squares = 0.0389 cm^2^) between the measurement data and calculated points, which is consistent with a 0.03 cm uncertainty in the measured *R*_80–20_ data.

Utilizing these results, it is possible to correlate matching criteria comparing *FWHM* with the clinical value of 0.1 cm for *R*_80–20_, i.e.,

(5) $$\Delta \mathrm{FWHM}=\Delta R_{80-20}{\left|\frac{dR_{80-20}\left(PME,FWHM\right)}{\mathrm{dFWHM}}\right|}^{-1},$$

(6) $$=\Delta R_{80-20}{\left|c_{1}\cdot \frac{R_{80-20}\left(PME,0\right)}{\mathrm{PME}}\right|}^{-1},$$

which results from the derivative of eq. (3) being substituted into eq. (5). For *PME* = 7, 13, and 20 MeV, Δ*FWHM* = 1.90, 2.16, and 2.03 MeV, respectively. Hence, a difference in agreement of 0.1 cm in *R*_80–20_ corresponds to an agreement of approximately 2.0 MeV in *FWHM*.

## 4 SUMMARY AND RECOMMENDATIONS

Based on the results of the present study, we conclude that a lightweight, permanent magnet spectrometer^1^ is a useful tool for measuring energy spectra of matched therapeutic electron beams, allowing their comparison and evaluation, both qualitatively and quantitatively. Comparison of energy spectra for all beams on a single accelerator in most cases showed that the *PME* and *FWHM* of the energy spectra did not always smoothly vary monotonically with beam energy, as otherwise expected. If improperly tuned, the accelerator produced a beam energy spectrum with an inappropriate value for the peak mean (*PME*) energy; also, suboptimal tuning of recirculated RF power can broaden the spectrum from its minimal *FWHM*.^7^ Suboptimal tuning was clearly visible in the shapes of energy spectra within the set for individual accelerators, which was supported by metrics such as *PME* and *FWHM*. Also, a comparison of energy spectra for a single beam energy on multiple matched machines showed unacceptably large variations in *PME* and *FWHM*.

Results of the present study correlated energy spectra metrics with PDD metrics for all seven energy beams on the six matched radiotherapy accelerators. Because of tolerances in initial beam matching, daily QA tolerances for single beam energies, and there being seven different energies on each machine, there was sufficient spread in the data to allow potential QA criteria for energy spectra metrics to be extracted. For beam matching at our institution, a matching criterion of 0.05 cm for *R*_50_ corresponds to 0.12 MeV for *PME*, and a criterion of 0.1 cm for *R*_80–20_ corresponds to 2.0 MeV for *FWHM*. For ongoing QA, the AAPM recommendation of ±0.2 cm for *R*_50_ corresponds to 0.48 MeV for *PME*. Changes in energy spectra metrics of this magnitude are easily measured using the magnetic spectrometer, which has approximately 0.1 MeV precision.

This study demonstrates the potentially increased sensitivity for beam matching when using the magnetic spectrometer to measure energy spectra (*PME* and *FWHM* metrics) in lieu of measuring PDD curves. Therefore, we recommend that the first machine, which becomes the reference machine for beam matching, be tuned to have properly spaced *PME* values and narrow *FWHM* values, both which vary smoothly and continuously with beam energy, and R_90_ values within 0.05 cm of specified values. Then, subsequent machines could be tuned to have *PME* values within 0.12 MeV and *FWHM* values within 0.5 MeV, which should produce matched *R*_80–20_ values within 0.05 cm and *R*_50_ and *R*_90_ values within 0.05 cm.

In conclusion, results of this study suggest that the lightweight, permanent magnet spectrometer used in this study could be a useful beam‐tuning instrument for the accelerator engineer to (a) match electron beams prior to beam commissioning, (b) tune electron beams for the duration of their clinical use, and (c) provide estimates of central‐axis percent depth‐dose (PDD) metrics following machine maintenance. However, a real‐time version of the passive lightweight, permanent magnet spectrometer used in this study is required to be practical. Although scintillating strips have been used in a real‐time version by Gahn et al.^19^ for laser‐plasma applications, a more compact detector, such as an amorphous silicon flat panel detector, is needed.

## CONFLICT OF INTEREST

The authors declare no conflict of interest.
